# Freeze–thaw recycling for fiber–resin separation in retired wind blades

**DOI:** 10.1038/s44172-025-00490-7

**Published:** 2025-08-14

**Authors:** Khalil Ahmed, Xu Jiang, Ghazala Ashraf, Xuhong Qiang

**Affiliations:** 1 College of Civil Engineering, Tongji University, Shanghai, China; 2 Department of Chemistry, Fudan University, Shanghai, China

**Keywords:** Civil engineering, Environmental impact

## Abstract

The disposal of decommissioned wind turbine blades represents a growing economic loss and environmental concern due to the non-recovery of durable glass fiber–reinforced epoxy composites. Existing thermal and chemical recycling methods often require high temperatures and toxic chemicals, causing material degradation. Here, we present a novel freeze–thaw-based method for fiber–resin separation as an alternative. The process uses only water at human-safe temperatures, leveraging ice-induced expansion to disrupt the glass fiber–epoxy interface. Microscopic imaging and weight analysis revealed visible interface separation, with three-dimensional imaging showing a ~ 65% increase in crack volume and a ~ 32% rise in connected porosity after freeze–thaw treatment. Glass fibers retained up to 96% of their original mechanical properties, demonstrating minimal structural damage. Microplastics were easily removed through filtration, and the effluent water remained near-neutral with low organic carbon levels, meeting global water safety standards. These findings highlight freeze–thaw cycling as a sustainable route for efficient fiber–resin separation with minimal environmental impact.

## Abstract (web summary)

Khalil Ahmed and colleagues propose a simple fiber–resin interface separation method for composites of retired wind blades using only water at human-safe temperatures. The method offers great promise for composite recycling across multiple industries.

## Introduction

Over the past two decades, wind turbines have been widely deployed as a cornerstone of global sustainability efforts. Wind blades, primarily made from glass fiber-reinforced epoxy (GRE) composites, are engineered with strong fiber–resin interfaces to ensure long-term durability^1^. However, after 20–25 years of service, environmental aging and fatigue-induced interfacial micro-cracking necessitate their decommissioning. These tightly bonded glass fiber-epoxy (GF-epoxy) composites are difficult to separate, and as a result, retired blades are often classified as non-recyclable material and are therefore landfilled despite their huge economic benefits^2^. Wind blades installed 20–30 years ago have begun reaching end-of-life, leading to a growing wave of decommissioned blades by 2025. Estimates suggest that 43 million tonnes of retired blade material will accumulate by 2050, creating an alarming challenge for managing such a vast volume of retired composites^3^.

Among various end-of-life (EoL) strategies for decommissioned blades, recycling is widely regarded as the most circular and sustainable, option due to its ability to recalim glass fibers and resin for potential reuse^4–6^. Current thermal and chemical methods achieve GF-epoxy separation by breaking the epoxy’s C–O bonds at elevated temperatures or through aggressive chemical treatments^7–10^. While effective in separating the glass fibers and epoxy, these methods are associated with significant drawbacks, including glass fiber degradation, epoxy dissolution, high energy demand, toxic emissions, and complex wastewater management^2,7,11,12^. For instance, pyrolysis and acid/base-catalyzed solvolysis can generate effluents with extreme pH and total organic carbon (TOC) values that exceed safe discharge thresholds, necessitating additional treatment and limiting industrial scalability^13–15^. For instance, thermal recycling via pyrolysis typically operates at ≥450 °C and produces off-gases (e.g., NO*ₓ*, SO*ₓ*, CO) and viscous pyrolysis oils with high TOC^16,17^, while also reduces glass fibers mechanical properties due to damaged glass fiber surfaces caused by thermal oxidation^8,18,19^. Chemical recycling, on the other hand, often uses strong acids or bases (e.g., H₂SO₄, NaOH) that yield highly acidic or basic effluents with high organic content, requiring costly pH neutralization and solvent recovery^7,10,20,21^. These environmental burdens and post-treatment steps reduce the feasibility of large-scale deployment^22,23^.

Here, we introduce a novel, environmentally friendly, cost-effective, and technically feasible freeze–thaw method that relies solely on ice and water at human-tolerable temperatures to efficiently separate GF–epoxy in decommissioned wind blades. The present work, for the very first time, strategically repurposes the pre-existing micro-cracks in decommissioned blades for targeted fiber–resin separation in GRE composites. The method’s performance is evaluated through multi-modal characterization, including interfacial separation efficiency (SEM, weight change, micro-CT), material integrity (FTIR, EDS, TGA), retention of basic nano-mechanical properties of glass fibers, and environmental safety of this approach via pH, TOC, and microplastic content of effluent water, which are critical factors in the real-world adoption of any recycling approach. This work focuses solely on GF–epoxy interface separation, with full fiber reclamation planned in the future through multi-phased recycling frameworks of pre-weakened GRE composites.

Here, we propose a novel freeze–thaw cycling method to separate fiber and resin in glass fiber-reinforced epoxy (GRE) composites by harnessing the unimaginable power of water at temperatures comfortably bearable to humans. Our method is inspired by the natural process of rock-splitting through freeze–thaw weathering. In nature, water enters pre-existing micro-cracks in rocks, expands as it freezes during cold cycles, and exerts pressure at the boundary area that propagates the cracks, ultimately leading to material fracture. This same principle can be utilized to de-bond fiber-resin interfaces in GRE composites.

Wind turbine blades, which must be retired after their service life due to fatigue, typically exhibit micro-cracks at or near the interface between the glass fibers and epoxy resin^10^. Although these micro-cracks are not large enough for complete failure of the structure (Supplementary Note 2, Fig. S2), they are sufficient enough to provide a channel for water ingress into them. Moreover, these blades are designed to be lightweight, with some voids intentionally created during the composite manufacturing process using foaming agents or other techniques, while others may form unintentionally due to poor manufacturing practices. These micro-cracks, along with voids and other structural defects formed during operation, can be effectively harnessed for fiber-resin separation through freeze–thaw cycling.

**Fig. 1:**
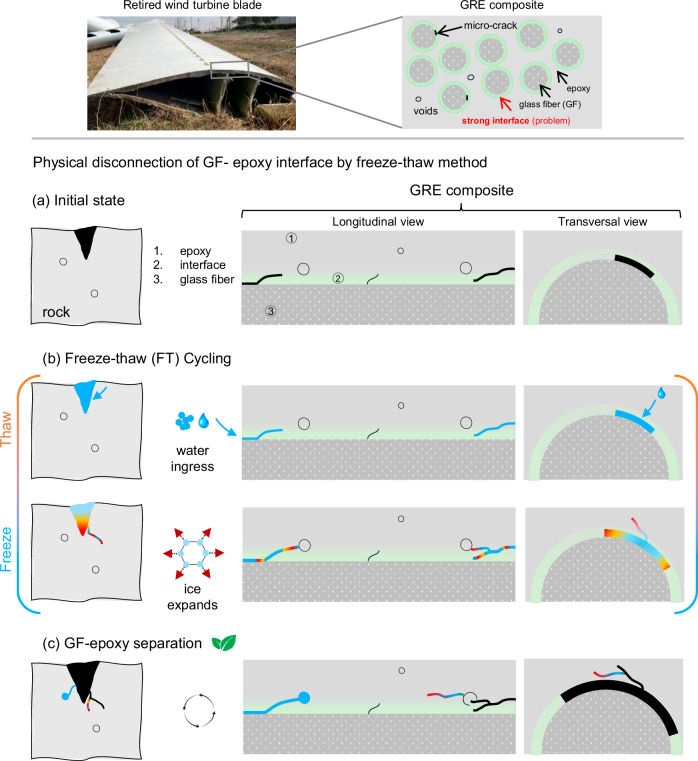
Research problem (strong GF–epoxy interface) illustrated at the top, followed by the mechanism of fiber–resin separation via freeze–thaw cycling, shown in longitudinal (middle) and transverse (right) views, with a rock-splitting example as a reference presented on the left. **a** Schematic showing pre-existing micro-cracks and voids in the composite structure. **b** Water penetration into micro-cracks during thawing, followed by expansion during freezing, which exerts pressure on the boundary walls, leading to the formation of new cracks. **c** Crack propagation and permanent fiber-resin debonding after repeated freeze–thaw cycles.

Here, we schematically demonstrate how this unique freeze–thaw approach works, as shown in Fig. 1. The freeze–thaw method works by infiltrating water into the pre-existing cracks and voids within the epoxy matrix during the thawing phase. Once the water has penetrated these defects, it expands during the freezing phase, exerting mechanical stress at the fiber-resin interface. With each freeze–thaw cycle, the cracks progressively propagate and expand, connecting with nearby voids. This chain reaction causes further stress concentrations, leading to more extensive cracking and eventual debonding of the glass fibers from the resin.

## Results and Discussions

### GF–epoxy separation mechanism and process efficiency

#### Scanning electron microscopic (SEM) analysis

**Fig. 2:**
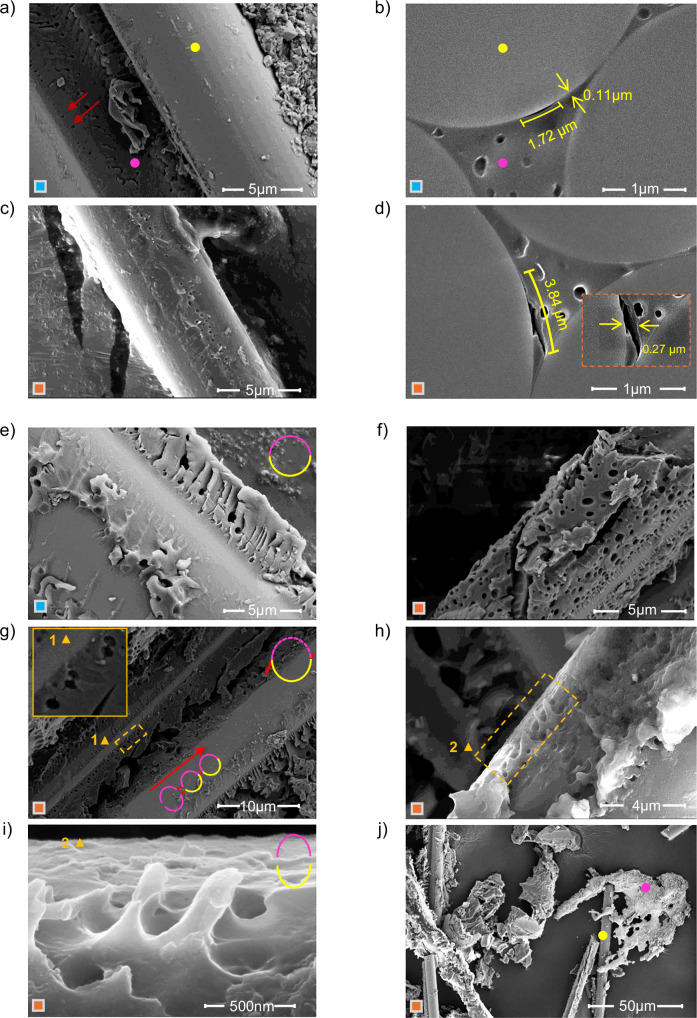
SEM analysis of the crack initiation and propagation in GRE composite leading to the fiber-resin interface separation after repeated freeze–thaw cycling. **a** Longitudinal view of the fiber-resin interface before freeze–thaw cycling, showing pre-existing voids and micro-cracks near the interface (indicated by red arrows). **b** Transversal view of the GRE composite, detailing the dimensions of pre-existing cracks. **c** Longitudinal view after freeze–thaw treatment, revealing widened and extended cracks at the fiber-resin boundary. **d** Transversal view showing further crack expansion, with inset magnifying the increased crack width. **e** Initial state of voids and micro-cracks near the fiber-resin boundary, with epoxy connected to glass fiber marked in yellow and unconnected epoxy in purple, forming complete circles, indicating an intact interface. **f** Early-stage crack formation in epoxy resin during freeze–thaw cycling. **g** Inset (triangle) shows micro-crack initiation in epoxy voids near the interface, with newly formed cracks highlighted in red. Crack propagation is demonstrated by half-split circles connected by new cracks, showing a chain reaction as water connects neighboring voids, creating pathways for further penetration. **h**, **i** Glass fiber surface showing remaining epoxy resin in an oval shape due to repeated pressure at the boundary. The triangle shows the enlarged area. **j** Final image showing complete separation of fiber and resin after freeze–thaw treatment.

The SEM micrographs provide a comprehensive analysis of the localized surface of the glass fiber-reinforced epoxy (GRE) composites interface before and after exposure to freeze–thaw cycling (Fig. 2a–d). Pre-thaw figures reveal the presence of pre-existing structural defects at or near the fiber-resin interface in longitudinal view (Fig. 2a), which are likely developed by the mechanical stress, environmental exposure, or manufacturing imperfections^24^. Although these defects are evident, they have not yet resulted in significant fiber-resin debonding, as the adhesive bond between the fibers and resin remains largely intact. A transverse view of the same GRE composite’s surface illustrates one of these micro-cracks which was measured as 1.72 µm in length and 0.11 µm in width (Fig. 2b) and indicate a potential weak point where moisture infiltration could occur during the freeze–thaw process. At this stage, the overall structural integrity of the composite seems to be well maintained.

After freeze–thaw treatment, SEM images show dramatic changes, with significant crack propagation and fiber-resin debonding (Fig. 2c, d). The longitudinal view clearly shows much longer and wider cracks propagating from the fiber-resin interface toward the outside (Fig. 2c). The transversal view reveals extensive cracks, now extending up to 3.84 µm in length and 0.27 µm in width, radiating from the fiber-resin interface and spreading outward (Fig. 2d). This propagation is driven by water penetration into micro-cracks during thawing and cyclical expansion during freezing^25^, and is further explained by crack propagation mechanism below.

#### Crack propagation mechanism

To understand the GF-epoxy separation mechanism further and validate our hypothesis, we applied the freeze–thaw treatment to shredded GRE composites (Supplementary Fig. S1e), which provided clear insights into crack initiation, propagation, and eventual fiber-resin separation (Fig. 2e–j). Initially, shredded glass fiber and epoxy resin show pre-existing voids and micro-cracks, which serve as water penetration points during the thawing phase (Fig. 2e). When water freezes and expands during the freezing phase, these sites become focal points for crack initiation and growth^26^, gradually separating the glass fiber from the epoxy matrix (Fig. 2f–j).

Before applying freeze–thaw cycles, glass fiber and epoxy remain largely bonded, though micro-cracks and voids are evident (Fig. 2e). Water infiltrates these cracks and voids during the thawing phase and expands during freezing phase leading to initial crack formation (Fig. 2f).

A comprehensive illustration of how these cracks propagate and can help fiber-resin separation in compact composite structures is shown in (Fig. 2g). The inset image shows small micro-cracks that have just appeared due to ice pressure at the void’s boundary walls, which are expected to propagate further in subsequent freeze–thaw cycles. With each freeze–thaw cycle, the newly formed cracks extend and connect with neighboring voids, creating a pathway for more water to penetrate. As a result, this crack continues to propagate, forming a chain reaction.

Semi-oval shapes of epoxy are seen adhering to the glass fiber surface, forming a sawtooth-like structure (Fig. 2h, i). This suggests that the continuous freeze–thaw process exerts increasing pressure on the boundary walls of the voids, altering their shape from circular to oval as they approach the point of splitting. The disappearing portions of the ovals indicate where epoxy has been removed due to the expansion of voids. This process continues and finally, the glass fibers and epoxy resin separate from each other over repeated freeze–thaw cycling (Fig. 2j).

### Weight change analysis

**Fig. 3:**
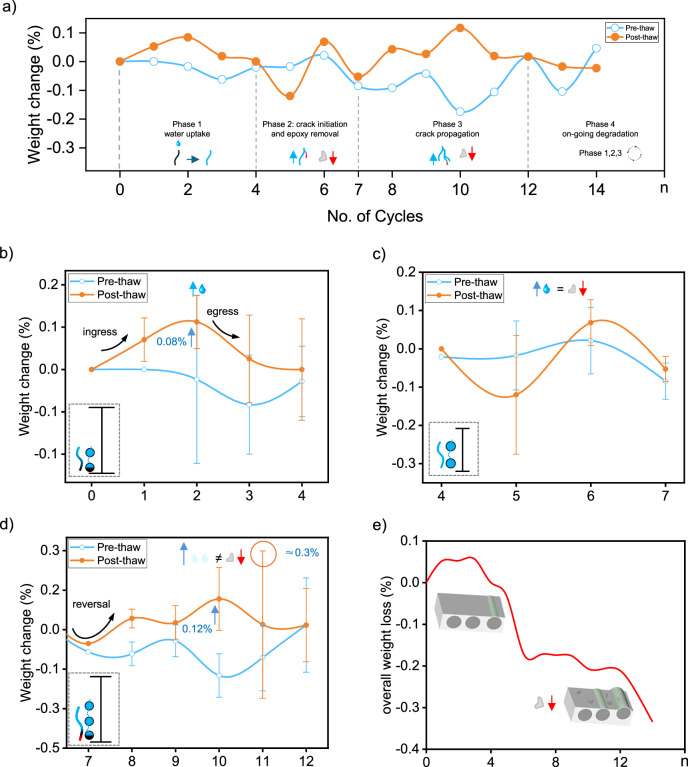
Progressive weight change in GRE composites under freeze–thaw treatment. Weight change (%) in glass fiber-reinforced epoxy (GRE) composites is shown over multiple freeze–thaw cycles (**a**), highlighting four distinct phases: Phase 1, water uptake; Phase 2, crack initiation and epoxy removal; Phase 3, crack propagation; and Phase 4, ongoing degradation. The pre-thaw (blue line) and post-thaw (orange line) curves illustrate a pattern of cumulative weight change, while the inset symbols illustrate mechanisms within each phase, such as water ingress, crack development, and epoxy loss. The detailed observation of each phase is shown from (**b**–**e**), where Phase 1 (**b**) shows water uptake, with larger error bars reflecting specimen variability; saturated and partially filled voids are depicted in the inset. Phase 2 (**c**) illustrates crack initiation as pre- and post-thaw curves intersect, indicating equilibrium between water uptake and epoxy removal. In Phase 3 (**d**), crack propagation is evident, with increased water ingress leading to overall weight gain; the inset shows crack extension reaching adjacent voids. Phase 4 (**e**) reveals ongoing epoxy degradation, with schematic diagrams indicating progressive resin loss. Pre-thaw refers to the weight measurement taken after a 30-min thaw following freezingPost-thaw refers to the weight measurement taken after an additional 8-h soak following that initial thaw.

The freeze–thaw treatment induces progressive weight changes in FRP composites as the number of cycles increases^27,28^. Moisture flow in porous materials like GRE composites typically involves three mechanisms: diffusion (ingress), effusion (egress), and capillary transport^29^. In glass fiber-reinforced epoxy (GRE) composites, this process was segmented into four phases based on weight changes observed pre- and post-thaw: Phase 1, water uptake; Phase 2, crack initiation and epoxy removal; Phase 3, crack propagation; and Phase 4, ongoing degradation (Fig. 3a). These phases reveal how successive cycles of water ingress, ice expansion, and thaw-induced stress progressively degrade the fiber-resin interface, leading to cumulative weight loss and structural compromise.

#### Phase 1: water uptake

During the early freeze–thaw cycles (Day 1–3), the GRE composite experiences a gradual weight increase due to water ingress into pre-existing microcracks and voids (Fig. 3b)^30^. On Day 2, this infiltration results in a 0.08% weight gain in the pre-thaw phase, with maximum water uptake reaching more than 0.10% of the initial weight. This initial phase of water absorption suggests substantial penetration into microcracks, promoting weight gain without significant structural degradation. The weight change curve indicates that pre-thaw weights are lower than post-thaw values across cycles, likely due to the minor detachment of loosely bound surface epoxy, a similar mechanism found in concrete ageing^31^. The periodic expansion of absorbed water during subsequent cyclic freezing and thawing exerts pressure on the fiber-resin interface, leading to further water uptake. However, this additional absorption is partially counterbalanced by the loss of surface epoxy, resulting in a minor net weight reduction^31^. Notably, large variations in weight are observed across all specimens during Phase 1, for both pre- and post-thaw measurements. Phase 1 thus reflects a dynamic process, involving both water ingress and egress driven by localized porosity and water-holding capacity rather than being a unidirectional process (ingress only). According to prior findings by Kakanuru^29^, when maximum moisture ingress and egress are nearly identical pre- and post-thaw, it suggests no significant material degradation, indicating that major epoxy detachment has not yet begun. At this stage, the composite primarily functions as a water-absorbing matrix.

#### Phase 2: crack initiation

Following the initial water uptake, Phase 2 shows weight fluctuations due to ongoing structural adjustments (Fig. 3c). On Day 5, the post-thaw weight decreases by approximately 0.15% relative to the original weight, while the pre-thaw weight nearly matches the initial value. This notable post-thaw reduction suggests the onset of epoxy detachment^32^. As cycles progress, these weight fluctuations gradually stabilize around Days 6 and 7, indicating that the composite is adapting to the freeze–thaw process and approaching an equilibrium where weight changes become more consistent.

During this phase, water ingress continues alongside epoxy detachment, as water penetrates newly formed cracks and exposed voids. However, a net weight reduction persists, as the material does not fully regain its pre-thaw weight due to ongoing epoxy loss. The standard deviation of weight change in this phase is lower than in Phase 1, suggesting that water has likely saturated all accessible micro-pores and voids, leading to more uniform weight changes across samples^33^. This uniformity indicates that the material is nearing saturation, ready for further crack propagation as freeze–thaw cycles continue.

#### Phase 3: crack propagation

As freeze–thaw cycling advances, Phase 3 (Fig. 3d) marks a critical shift, with the post-thaw weight curve showing a sharp upward trend, reaching up to 0.12%. This indicates intensified water ingress outpacing epoxy loss, resulting in a net post-thaw weight gain^29^. The expanded network of saturated cracks and voids from Phase 2 now enables additional water infiltration, initiating a chain reaction of water absorption.

Capillary action along the fiber-resin interface may now play a more pronounced role, drawing water further into microcracks or interfacial voids formed in earlier cycles^33^. This effect likely creates localized areas of high-water absorption, particularly where fiber orientation favors capillary movement, amplifying the observed weight gain. A marked decrease in pre-thaw weight (−0.15%) is also observed, likely due to advancing crack propagation and the detachment of composite fragments. The widening of cracks through repeated cycles reduces the composite’s ability to retain water, allowing water to drain toward the surface and potentially escape during the pre-thaw stage. In smaller cracks, capillary forces help retain water through surface tension. However, as these cracks widen, capillary forces weaken, allowing trapped water to escape more easily. These capillary movements of water molecules, can push liquid water outward, reducing the composite’s potential for post-thaw weight gain^34^.

The variability in weight change remains significant, with a peak standard deviation of 0.3% on Day 11, reflecting the heterogeneity seen in Phase 1. This suggests that as initial cracks saturate, new cracks and voids emerge with continued crack propagation. Phase 3 thus represents an active period of structural evolution, where advancing cracks promote further water ingress and composite degradation.

#### Phase 4: on-going degradation

In this phase (Fig. 3e), the weight decrease becomes more pronounced with each cycle, as the composite’s structural integrity further deteriorates. The fluctuating pattern of temporary water gain and persistent material loss suggests a history-dependent diffusion process^35^, where previous cycles have left the composite increasingly vulnerable to water ingress while simultaneously reducing its overall mass. Floating debris, likely epoxy fragments, was observed during this phase, indicating material degradation. This behavior provides insight into the degradation mechanisms at work, revealing that, by Phase 4, the composite has transitioned from a state of relative stability to one of progressive cracking under continued freeze–thaw cycling.

### Micro-CT analysis

**Fig. 4:**
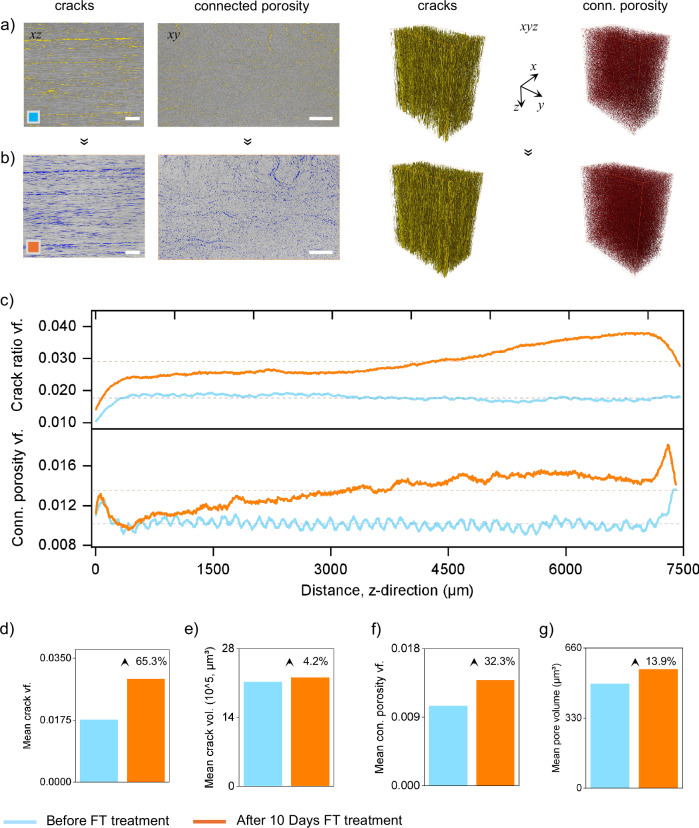
Micro-CT analysis of internal GF-epoxy separation evolution in GRE composites before and after days of freeze–thaw (FT) cycling. **a**, **b** 2D longitudinal and transverse cross-sections showing cracks and connected porosity, along with 3D volumetric reconstructions of their spatial networks. The untreated sample (**a**, blue box) displays few and unconnected cracks and pores, whereas the FT-treated sample (**b**, orange box) reveals a denser, wider and more continuous network of internal cracks and pores. **c** Spatial profiles of crack ratio and connected porosity along the specimen length (Z-direction), showing both end-region amplification and core-region degradation after FT treatment. **d**–**g** Quantitative bar plots comparing: (**d**) mean crack volume fraction, (**e**) mean crack volume, (**f**) connected porosity volume fraction, and (**g**) mean pore volume before and after FT treatment. Pictograms depict hypothesized interface degradation mechanisms supported by these results. The schematic below defines end regions (0–300, 7100–7400 µm) and core region (300–7100 µm) of tested GRE composite specimen.

To quantitatively validate the fiber–resin separation mechanism previously observed through SEM and weight change analyses, micro-computed tomography (micro-CT) was conducted on GRE composites before and after ten days of freeze–thaw (FT) treatment. This non-destructive, high-resolution technique provided three-dimensional insights into internal crack propagation and porosity which are key indicators of interface separation^36^. The 2D and 3D micro-CT renderings (Fig. 4a, b) visually emphasize these transformations. The 2D longitudinal and transverse slices demonstrate a substantial increase in visible micro-cracks and pore networks after FT treatment. In particular, the 2D transverse view shows a marked increase in connected porosity after freeze–thaw treatment, reflecting the lateral merging of previously isolated voids. Similarly, full-volume 3D reconstructions of cracks and connected porosity provide a comprehensive visualization of internal degradation. These renderings reveal a densified crack network and a highly interconnected porosity structure post-treatment, in comparison to the limited and discontinuous features in the untreated sample.

#### Crack propagation and porosity distribution

To find out the spatial distribution of GF-epoxy separation, we evaluated the layer-by-layer profiles of cracks and porosity along the specimen’s Z-direction (fiber axis), focusing on crack volume fraction and connected porosity which is shown in Fig. 4c. From the spatial profiles of cracks and porosity, two primary regions were identified: (i) End Regions (ER, 0–300 µm and 7100–7400 µm), and (ii) Core Region (CR, 300–7100 µm). Before FT treatment, the uniform crack and porosity distribution across the specimens’ core region reflects the consistent quality of molding used in modern wind blade manufacturing^37^. However, after FT treatment, both metrics showed a clear increase across the specimen, with the most pronounced changes occurring at one end. Here, the crack volume fraction increased by over 110%, (~0.038), while connected porosity rose by over 50% (~0.0186). This end-region amplification indicates a strong influence of water ingress in initiating edge-localized damage. The rest of the specimen exhibited a more gradual and steady increase in both crack and porosity metrics, particularly within the CR, indicating progressive degradation from freeze–thaw cycling. Although not the main focus of this study, the asymmetric damage observed at the ends likely reflects differences in surface preparation or cutting quality^38,39^. These inconsistencies may have affected local permeability and accelerated crack growth in more vulnerable areas^40^. Such findings highlight that surface engineering may be used to either suppress or strategically promote interface separation, depending on the recycling goal which, otherwise, challenging to achieve with thermo-chemical methods.

#### Quantitative analysis of GF–epoxy separation efficiency

Following the spatial observations, a quantitative analysis was conducted to assess the degree of interface degradation more precisely. Four key parameters: (i) mean crack volume fraction, (ii) mean crack volume, (iii) mean connected porosity volume fraction, (iv) and mean pore volume were extracted from the micro-CT data. Their numerical comparison is shown in Table 1, with graphical representations in Fig. 4d–g.

**Table 1:** Quantitative comparison of internal structural metrics in GRE composites before and after freeze–thaw treatment

Parameter	Before FT treatment	After 10 days FT treatment	% Change
Mean crack volume fraction	0.0176	0.0291	65.3↑
Mean crack volume (µm3)	2120851	2210353	4.20↑
Mean connected porosity volume fraction	0.01048	0.01385	32.3↑
Mean pore volume (µm3)	491.86	560.29	13.9↑

The mean crack volume fraction increased from 0.0176 to 0.0291 after FT treatment, indicating a 65.3% rise (Fig. 4d). This substantial increase supports the crack propagation trends visualized earlier in SEM, micro-CT 2D, 3D images. In contrast, the mean crack volume only increased by 4.2%, suggesting that while cracks proliferated, they remained narrow (Fig. 4e). This modest increase is likely due to cyclic freeze-induced pressure that caused microcrack widening rather than large-scale structural failure. Similarly, connected porosity volume fraction rose by 32.3% (from 0.01048 to 0.01385), while mean pore volume increased by 13.9% from 491.86 to 560.29 µm³ (Fig. 4f, g). This supports the earlier interpretation that repeated freeze–thaw cycles enlarged pre-existing voids and connected isolated pores into a continuous network. Such crack-bridging behavior was previously hypothesized in SEM and weight change analyses, while visually identified in Fig. 4a, b, but is now quantitatively confirmed through micro-CT. The transition from isolated to connected voids plays a critical role in forming sustained pathways for water ingress and crack growth^41^.

Overall, these results confirm the effectiveness of freeze–thaw cycling in promoting GF–epoxy interface separation. Importantly, the findings reinforce that the method’s primary function is not full composite disintegration but selective interface debonding. However, this targeted interface separation can facilitate efficient glass fiber recovery via mechanical processing in multi-phase recycling frameworks in future.

### Material characterization

#### Elemental composition analysis, (EDS)

**Fig. 5:**
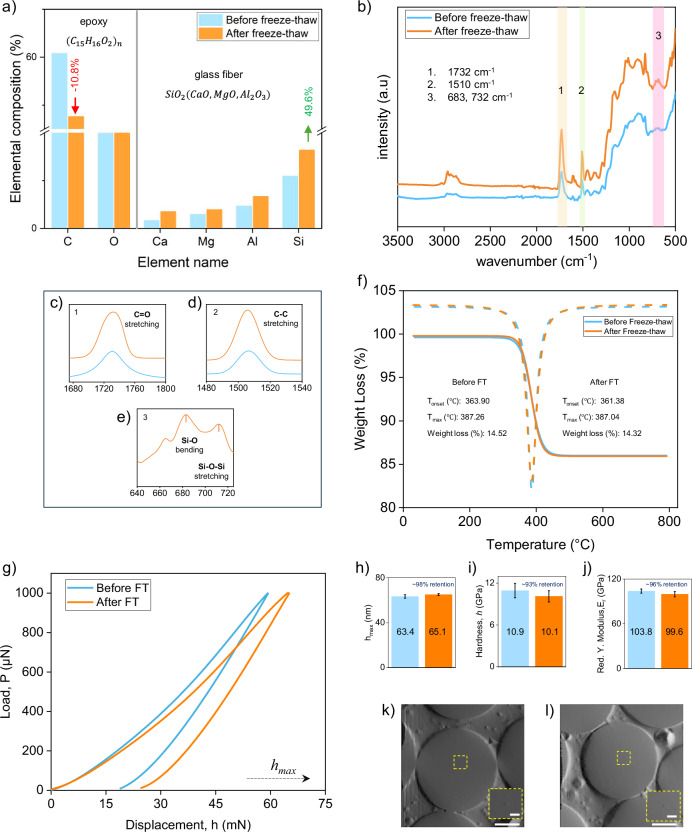
Elemental, chemical, thermal, and mechanical assessment of GRE composites before and after freeze–thaw treatment. **a**–**e** Elemental composition, FTIR spectra and key bond regions of glass fibers (**f**) TGA curves of GRE composites. **g**–**l** Nanoindentation results: (**g**–**j**) load–displacement, penetration depth, hardness, and Young’s modulus. **k** In situ SEM image of a fiber before treatment, showing a visible indent location (inset) and partial interface de-bonding. **l** Post-treatment fiber with no visible surface damage but more pronounced de-bonding at the fiber–resin interface.

Elemental analysis using EDS provided insight into compositional shifts in the GRE composite after freeze–thaw cycling, crucial for recycling applications. Notably, the carbon content, a primary constituent of the epoxy resin^42^, decreased from 60.42% to 53.84% whearas, the elemental composition of oxygen (O) remained nearly identical (~29%) following the treatment (Fig. 5a). This interesting phenomenon could be explained by the GF-epoxy separation process as explained earlier in Fig. 2e–j. During the freeze–thaw process, water penetrates and expands within pre-existing cracks and voids at the GF-epoxy interface (Fig. 2e–j), causing localized epoxy detachment. Since carbon and oxygen are the dominant elements in epoxy resin, this detachment leads to a reduction in the measured carbon content as well as oxygen. While oxygen from the epoxy resin (e.g., C–O, C = O functional groups) is lost along with the detached epoxy, more of the glass fiber’s surface (i.e., oxygen from the SiO₂ structure) becomes exposed. This increased exposure of oxygen from the underlying glass fibers compensates for the oxygen lost from epoxy detachment, thereby maintaining an almost constant overall oxygen content. In contrast, glass fibers contain no carbon, which means there is no compensating source for the lost carbon. As a result, a net reduction in the overall carbon, while near constant oxygen content is observed.

Simultaneously, the treatment increased the visibility of silicon (Si), an essential component of glass fibers, by 42.86%, as well as aluminum (Al), magnesium (Mg), and calcium (Ca), elements often present as strengthening agents in the composite structure For instance, Al content rose from 2.37% to 3.38%, Mg from 1.49% to 2.0%, and Ca from 0.87% to 1.79% as shown in Table 2. These compositional shifts suggest that freeze–thaw cycling effectively detaches the epoxy matrix from the glass fiber surface, exposing structural elements previously obscured by the resin layer. In comparison to current chemical and thermal treatments, which often compromise glass fiber structure and cause leaching effects^22,23^, the freeze–thaw approach preserves the integrity of glass fibers while promoting efficient separation which supports sustainability.

**Table 2:** Elemental composition of GRE composites before and after freeze–thaw (FT) treatment

Elemental composition (%)										
Element Name	C	O	Na	Mg	Al	Si	Cl	K	Ca	Fe
Before FT	60.42	29.06	0.14	1.49	2.37	5.50	0.13	–	0.87	–
After FT	53.84	29.67	0.20	2.00	3.38	8.23	0.22	0.21	1.79	0.30

#### Functional groups analysis (FTIR)

The FTIR spectra revealed the presence of characteristic C-H stretching peaks (2800-3000 cm⁻¹) in both pre- and post-treatment samples, confirming the preservation of the hydrocarbon structure that forms the epoxy resin’s backbone (Fig. 5b)^42^. The increased intensity observed in the peaks at 1732 cm⁻¹ and 1510 cm⁻¹, corresponding to ester C = O stretching and C-C stretching in the epoxy resin, indicates that these functional groups became more exposed following freeze–thaw treatment (Fig. 5c, d). This exposure suggests a physical separation of the resin from the fiber surface, rather than a chemical breakdown, highlighting the resin’s potential for immediate reuse in secondary applications.

Additionally, the emergence of new peaks at 712 cm⁻¹ (Si–O bending) and 664.5 cm^−1^ (Si–O–Si stretching) post-treatment points to increased exposure of the silicate network inherent to glass fibers (Fig. 5e), further confirming the selective displacement of the epoxy matrix from the fiber surface. These findings suggest that freeze–thaw cycling effectively separates the fiber-resin interface, promoting recyclability by enhancing material separation while preserving the chemical structure of the resin. This approach contrasts with harsh thermal and chemical treatments, which often degrade epoxy resin, making it unsuitable for reuse. By maintaining the structural integrity of the epoxy, the freeze–thaw method not only supports sustainable fiber-resin separation but also adds value to the recovered resin, potentially enabling its use in secondary applications with minimal further processing.

#### Thermogravimetric analysis (TGA)

Thermogravimetric analysis (TGA) was performed to evaluate the thermal stability and decomposition patterns of the GRE composite before and after freeze–thaw treatment (Fig. 5f). The three-stage mass loss pattern observed in both treated and untreated samples indicates that the process does not alter the bulk thermal properties^32^, with onset decomposition temperatures around 361–364 °C and maximum decomposition near 387 °C^43^. The negligible variation in weight loss (14.528% before and 14.323% after treatment) further confirms the selective nature of freeze–thaw cycling, targeting only the interface while preserving the material’s thermal integrity.

#### Nano-mechanical characterization of glass fibers

The mechanical properties of glass fibers within the GRE composite were evaluated using in situ nanoindentation before and after freeze–thaw (FT) treatment (Table 3, Fig. 5g–l). The load–displacement curves (Fig. 5g) revealed a slight increase in penetration depth following FT treatment. Specifically, the maximum indentation depth, ${h}_{\max }$ increased by approximately 2.6%, from 63.4 nm before FT to 65.07 nm after FT (Fig. 5h). Nanoindentation hardness showed a small decline of approximately 7.58%, from 10.9 GPa to 10.1 GPa (Fig. 5i), whereas the reduced modulus, ${E}_{r}$, decreased from 103.8 GPa to 99.64 GPa, corresponding to a ~ 4% reduction (Fig. 5j). Despite these slight changes, the retention of mechanical properties remained high, with ~98% retention in ${h}_{\max }$ related deformation capacity, ~93% retention in hardness, and ~96% retention in elastic modulus after FT cycling.

**Table 3:** The nano-mechanical properties of the glass fibers before and after separation by freeze–thaw method for GF-epoxy separation method in retired wind blades

Parameters	Before FT	After FT	% Change
Penetration depth, *h*_*max*_ (nm)	63.40 ± 1.55	65.07 ± 0.88	+2.63↑
Hardness, *H* (GPa)	10.94 ± 1.03	10.11 ± 0.81	−7.58↓
Reduced Young’s Modulus, *E*_*r*_ (GPa)	103.88 ± 2.85	99.64 ± 3.53	−4.08↓

A slight reduction in the nano-mechanical properties of glass fibers was observed following freeze–thaw (FT) treatment, which can be attributed to a combination of surface- and interface-related effects. Glass fibers are known to develop nano- and micro-scale surface defects during manufacturing, prolonged fatigue loading in service^24^, and specimen preparation processes such as cutting and polishing^39^. It is considered that these pre-existing flaws were likely aggravated by repeated cyclic thermal stresses^40^. Moreover, prolonged preservation of GRE composites immersed in water at low temperatures, −30 °C for ~16 h every day, and the sharp thermal gradients generated by the immediate transfer of specimens from −30 °C to +50 °C may have further induced localized stresses, thereby leading to glass fibers surface cracking and minor structural weakening^44^. Additionally, it is considered that this loss of interfacial support likely led to greater localized deformation during indentation and contributed to the modest reductions observed in the overall mechanical properties^45^. This interface splitting was previously observed in scanning electron microscopy and micro-CT analyses, and is further confirmed by the present in-situ digital images captured during nanoindentation testing (Fig. 5k, l). Finally, although careful control of machine parameters and fiber selection was maintained, minor sources of variability such as calibration shifts, surface contamination, or small variation in fiber’s diameter cannot be entirely excluded, particularly due to the sensitivity of nano-mechanical measurements^46^.

Despite the slight reduction, the high retention of nano-mechanical properties, especially the elastic modulus (96%). These findings suggests that the freeze–thaw method largely avoids intrinsic deterioration of the glass fiber cores. Future optimization of specimen cutting techniques, surface polishing methods, and freeze–thaw cycling conditions is expected to further improve retention of basic mechanical properties.

### Microplastic release and post-treatment water quality assessment

**Fig. 6:**
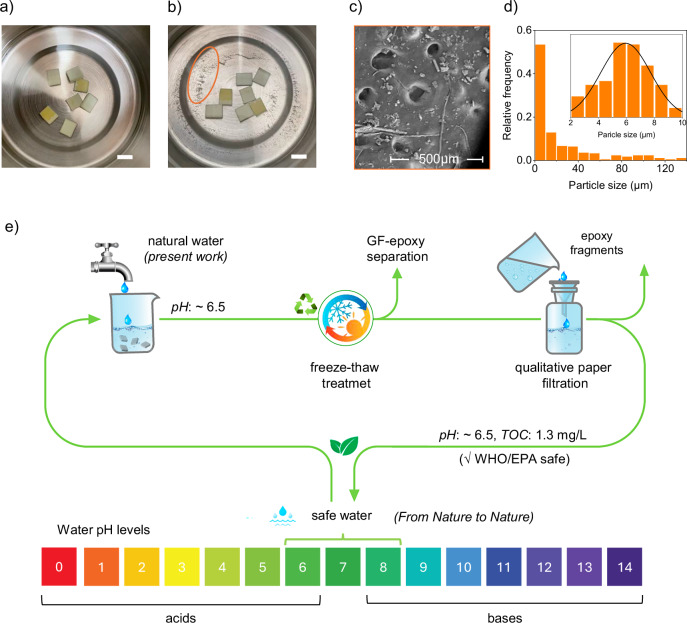
Microplastic release and environmental assessment of freeze–thaw recycling. **a**, **b** Visual inspection before and after treatment shows detachment of epoxy fragments from GRE surfaces. **c** SEM image of filtered residue reveals micrometer-scale debris; most particles are <40 µm, with some as small as 2 µm (inset). **d** Particle size distribution of filtered epoxy fragments. **e** Schematic of the freeze–thaw method using natural water (pH ~6.5) and post-treatment filtration (5 µm), yielding neutral-pH water with low TOC (1.3 mg/L), within WHO/EPA surface water limits^47,48^.

To evaluate potential environmental risks, we assessed the release of microplastics and effluent water quality following freeze–thaw treatment of GRE composites. The initial water used in this study, sourced from a drinking water facility, appeared visually clean with minimal detectable microplastic content (Fig. 6a). However, after freeze–thaw treatment, a considerable accumulation of white, powdery debris is visible at the base of the container (Fig. 6b). This debris was identified as detached epoxy resin from the localized surface of GRE composite specimens (Supplementry Note 3, Fig. S3). Although prior SEM–EDS and FTIR analyses showed no visible signs of elemental or chemical leaching from the GRE composites, it remained essential to thoroughly evaluate the effluent water quality. Therefore, we examined the epoxy fragments generated after freeze–thaw treatment. Particle size analysis from SEM images indicated that the fragmented epoxy microplastics ranged predominantly from 2 to 140 µm, with the highest frequency between 2–10 µm and moderate occurrences between 10–40 µm (Fig. 6c, d). The vast majority of these particles can easily be removed by using commercially available fine mesh filters with as little as 0.1 µm pore diameter which can be expected to be sufficient to filter out these particles.

Similarly, the filtered effluent water after GF–epoxy separation showed a pH of 6.38, which was close to the actual drinking water used in the study which has an initial pH of 6.46 (Fig. 6e). Additionally, TOC measurements showed that the filtered water contained only 1.3 mg/L of total organic carbon, which well aligns with TOC level of surface water, and is well below the WHO/EPA threshold for drinking water of ≤2 mg/L^47,48^. The marginal differences in water chemistry after freeze–thaw treatment can be attributed to minor instrumental variations or possible contamination. Additionally, it is considered that microplastic particles may have passed through the 5 µm pore size filter paper used in this study, as SEM images revealed several particles smaller than 5 µm, with the smallest observed particle measuring approximately 2 µm (Fig. 6d, inset). These extremely small particles could however, easily be filtered out by integrating multi-stage filtration process, including fine-mesh sieves, activated carbon filters or other advanced physical filtration techniques^14^. Nevertheless, both, the pH level and TOC level of effluent water used in the present study remained well aligned with WHO/EPA standards for safe water (pH: 6.5–8.5, TOC: ≤2 mg/L)^47,48^. The near identical water chemistry of the influent and filtered effluent water used in the present novel recycling approach indicate the minimal effect of this unique approach to the nature.

### SWOT analysis of freeze–thaw method in comparison to thermo-chemical methods

The freeze–thaw method presented in this study offers a fundamentally different approach to glass fiber–epoxy (GF–epoxy) separation than conventional thermal and chemical recycling techniques. Below are its comparative advantages and potential opportunities.

#### Core techno-environmental advantages of freeze–thaw method

The most significant aspect of the freeze–thaw technique is that the method precisely targets the main bottleneck issue i.e. the interface, without significantly affecting the glass fibers, which has the potential for reuse with minimal treatment. For instance, as demonstrated in the present study, ~65% increase in crack extension and a ~32% increase in connected porosity were observed after FT cycling, while the structural integrity of the glass fibers and epoxy as confirmed by EDS, FTIR, and TGA analyses remained nearly unaffected. In contrast, thermal and chemical methods, although they can achieve nearly 100% fiber-resin separation, they typically attack the bulk matrix, leading to uncontrolled degradation of both resin and fiber^5,8,10^ requiring extensive post-recycling treatments^23,49^. Although greener, lower-temperature thermo-chemical methods have been proposed^5,10,20,50^, their reliance on rare and costly metals poses scalability challenges and limits widespread application.

In addition, nano-mechanical characterization confirmed that glass fibers retained approximately 93–96% of their original properties after 10 days of freeze–thaw cycling, indicating minimal damage to the structural core. This retention could be further improved through optimized freeze–thaw conditions in future studies. Although direct comparison with fully reclaimed glass fibers was not possible in the present study, nano-mechanical characterization of embedded fibers provides a reliable estimate of their mechanical integrity before and after freeze–thaw conditioning. Our findings are consistent with previous studies reporting retention rates above 90% under similar conditions^51,52^. Such levels of mechanical retention in thermally or chemically recycled glass fibers are usually achieved through post-recycling glass fiber’s surface treatments. These post-processing steps add complexity and introduce additional environmental and economic burdens^22,23^. Furthermore, from an economic perspective, the freeze–thaw method offers substantial advantages. The process requires only basic freezing equipment and water, eliminating the need for solvent recovery systems or high-temperature reactors. Since the same water can be reused with minimal filtration and returned safely to natural sources, overall process costs remain relatively low.

The most significant aspect of the freeze–thaw method lies in its minimal environmental footprint and operational simplicity. The freeze–thaw method operates at human-bearable temperatures, eliminates the need for chemical solvents, and uses only natural water as an influent while producing near-neutral effluent (pH ~6.36) with very low TOC (~1.3 mg/L), as observed in the present study. These values remain well within safe water discharge limits (pH: 6.5–8.5, TOC: ≤2 mg/L) set by WHO/EPA, and therefore require minimal wastewater treatment^47,48^. In comparison, thermal recycling processes usually operate at ≥450 °C and emit acidic off-gases and high-TOC pyrolysis oils, while chemical approaches involving acids or bases such as HCl, KOH, or nitric acid etc. produce extreme pH effluents (typically pH: ≤4 or ≥9) with TOC levels frequently reaching thousands ofmg/L^7,13^. These waste streams demand intensive water neutralization and create secondary environmental and economic concerns, which the freeze–thaw process inherently avoids.

#### Current Limitations and Strategic Considerations

While the freeze–thaw method shows great potential, its slower processing time and reliance on fiber–matrix interface properties pose initial challenges. These limitations, however, can be addressed by improving thermal cycling efficiency through physical modifications, such as composite cutting and surface preparation, which can enhance water permeability and promote more effective interface separation^39,40^. Furthermore, the interface separation efficiency can be further enhanced by the introduction of thermally expandable nano-particles^53^. Also, although the pre-existing micro-cracks, which are limited in number (Supplementary Note 2, Fig. S2), facilitate early-stage separation, they are not strictly necessary for the freeze–thaw process to be effective. Over time, the method can still induce progressive interface degradation independently, as observed in earlier studies where, even in the absence of visible interfacial cracks, repeated freeze–thaw cycling initiated interfacial weakening and micro-crack formation^25,26,35,52^. In addition, similar to thermo-chemical approaches, longer and more uniform glass fibers can be recovered by pre-cutting the composites to specified lengths. However, unlike thermo-chemical methods that rely on complex kinetic and chemical reactions to achieve fiber–resin separation, the freeze–thaw approach enables optimization through simpler physical parameters, such as customized surface treatments. This advantage makes the method particularly suitable for aged or decommissioned composites where interfacial fatigue is already present, such as stored wind turbine blades awaiting end-of-life processing. Moreover, freeze–thaw treatment may also serve as a preparatory step in broader multi-phase recycling approaches^50,54^. In terms of economic and environmental feasibility, while the freeze–thaw process involves some energy input for refrigeration, its overall demand might be considerably lower than that of thermal or chemical recycling. This, however, requires further validation through lifecycle assessments. Advances in green cooling systems, such as salt-based ionocaloric cooling, and the integration of renewable energy for cooling purposes^55,56^, offer promising routes toward making the process more sustainable.

The method also faces some threats, including the need for initial investment in industrial-scale freezers and the challenge of regulatory validation. Additionally, it must compete with well-established thermal and chemical recycling methods that are already widely adopted. Nonetheless, the unique strengths and potential opportunities of the freeze–thaw method (Table 4) position it as a promising and innovative alternative for large-scale recycling applications.

**Table. 4:** SWOT analysis of freeze–thaw method for fiber-resin separation in polymer composites

Strengths	Weaknesses
▲ *Eco-friendly:* Uses natural water at human-safe temperatures, which can be safely returned back to nature with minimal treatment. ▲ *High mechanical retention:* Preserves 93–96% of glass fiber properties, with further improvements possible by adjusting freeze–thaw parameters. ▲ *Physical de-bonding:* Effective fiber–resin separation can be achieved via water ingress and ice pressure, only. ▲ *Customizable interface separation:* Fiber-resin separation ratio can be customized through physical parameter, e.g. composite surface preparation. ▲ *High scalability:* With TRL 9, the method is immediately applicable to various polymer composites across industries.	▼ Longer processing time due to multiple freeze–thaw cycles and reliance on fiber-matrix interface properties. ▼ Limited industrial adoption as the method is still in its early stages.
Opportunities	Threats
√ Longer, uniform fibers can be recovered through pre-cutting composites to desired length. √ Especially suitable for decomissioned composites where degraded interfae already exists √ Can be used as pre-processing step in multi-phase recycling frameworks	× Initial investment needed for industrial scale freezer procurement. × Regulatory validation and competition with well-established thermal and chemical methods.

### Outlook and future prospects

This study presents a novel freeze–thaw method that enables targeted debonding of the GF–epoxy interface without the use of chemicals or high temperatures. While the current work focused on interface separation, future studies will explore integrating this approach as a pre-processing step in multi-phased recycling frameworks to facilitate efficient recovery of chopped glass fibers. Recovered glass fibers can potentially be reused in structural or non-structural composite applications such as automotive components or building materials. Furthermore, cross-linked epoxy resin fragments recovered through post-filtration in the present study and from future works, can be milled and repurposed along with other reinforcements such as glass fibers into fine aggregate in cement^57^, concrete^58^, multi-purposed panel boards^59^, wood–plastic composites, or even decorative products^60^. These combined strategies position the freeze–thaw^61–64^ method as a scalable, low-carbon solution with strong potential for full-component recovery in sustainable end-of-life (EoL) composite management.

### Conclusions

This study introduces a novel freeze–thaw cycling method to separate glass fibers and epoxy resin in GRE composites sourced from decommissioned wind turbine blades. In contrast to thermal or chemical processes that compromise material integrity and require harsh conditions, the freeze–thaw method enables interface separation through water ingress and ice expansion, while relying only on simple equipment and ambient-safe temperatures. Multimodal analyses using SEM, micro-CT, and weight change measurements confirmed progressive interface separation, with a ~65% increase in crack volume and a ~32% rise in connected porosity post-treatment. The structural and functional integrity of the materials was well preserved: EDS and FTIR revealed no chemical deterioration; TGA showed consistent thermal behavior; and nanoindentation testing demonstrated up to 96% retention of the glass fibers’ elastic modulus. Environmental assessment further validated the process’s safety, as the effluent water exhibited near-neutral pH, low TOC, and contained filterable epoxy fragments, all within WHO/EPA limits. Together, these findings establish freeze–thaw cycling as a scalable, environmentally benign strategy for GRE composite recycling. The method is particularly relevant for retired composites and offers strong potential for integration into multi-phase recycling frameworks aimed at full-component recovery and circular economy implementation in wind blade end-of-life management. Owing to its universally adaptable interface-driven fiber-resin separation mechanism, the method may also be extended to other fiber-reinforced composites used in the automotive, aerospace, and marine sectors.

## Methods

The retired wind turbine blade pieces containing glass fiber-reinforced epoxy (GRE) composites, were collected from a decommissioning site and processed into standardized specimens for experimental analysis. Two freeze–thaw (FT) treatments were conducted under identical thermal cycling conditions (ASTM D6944-15): a 3-week FT conditiong for interfacial weakening and crack development, and a separate 10-day FT treatment informed by earlier weight change data for microstructural and environmental characterization. Key analyses includes interfacial characterization (SEM, weight change monitoring, micro-CT), material integrity (FTIR, EDS, TGA), retention of basic nano-mechanical properties of glass fibers, and post-treatment water quality assessment (pH, TOC, and microplastics). Full experimental details and specimen preparation procedures are provided in Supplementary Notes.

## Supplementary information

Supplementary Information

## Data Availability

The datasets generated during and/or analyzed during the current study are available from the corresponding author on reasonable request.
