# Supplementary material for: Freeze–thaw recycling for fiber–resin separation in retired wind blades
Source: Commun Eng. 2025 Aug 14;4:153. doi: 10.1038/s44172-025-00490-7 (PMC12354729; doi:10.1038/s44172-025-00490-7)
Supplement: Supplementary file 1 — Supplementary Information [file 44172_2025_490_MOESM1_ESM.docx]

**Supplementary Note S1. Materials and Methods**

**Materials and Specimen Preparation**

***Material Collection***

Wind turbine blade waste pieces were collected from a decommissioning site in Shenyang County, Yancheng City, Jiangsu Province. Due to transport constraints, pre-cut pieces of manageable size were visually identified and selected on-site (Supplementary Figure S1a,b). These pieces were presumed to be glass fiber-reinforced epoxy (GRE) composites, though the exact material composition was unknown at this stage. Subsequent analysis was required to confirm the material composition before initiating experimental work. The selected blade segments were then transported to the laboratory for further processing.

***Composite Cutting and Specimen preparation***

The collected blade waste pieces were cut carefully by a diamond cutter to remove the unwanted materials such as balsa wood while retaining the areas with GRE composites. The GRE cut pieces were further scaled down into smaller specimens (~9 mm x 8 mm x 3 mm) for freeze-thaw conditioning. Due to cutting blade limitations, a dimensional variation of up to ±1.5 mm was observed in length. A few test specimens were selected and mechanically shredded in order to understand the crack propagation mechanism (Supplementary Figure S1c-e).

***Material Confirmation***

The material's composition was first confirmed from shredded GRE composites by using Elemental Dispersive Spectroscopy (EDS) analysis before the freeze-thaw treatment. The analysis identified carbon (C), oxygen (O), and silicon (Si) as the primary elements, with concentrations of 60.42%, 29.06%, and 5.50%, respectively (Table 2), which is consistent with the expected composition of glass fiber reinforced epoxy composite material for wind turbine blades [64]. After material’s confirmation, the effect of freeze-thaw treatment for eco-friendly GF-epoxy separation was studied as follows.

***Surface Polishing of GRE Specimens***

The GRE specimens were first cleaned with ethanol and laminated with a plastic sheet before being cast in reversible resin within a silicone mold, ensuring that surface entry channels for water ingress were not blocked by resin. The resin was cured under ultraviolet (UV) light and the specimens were then removed from the mold. One surface of each specimen, containing transverse fibers, was polished using progressively finer sandpapers (600, 1200, 2000, 3000, and 4000 grit) on an automatic polishing machine. After polishing, the specimens were heated in an oven at 80 °C to remove resin residue, followed by ethanol cleaning to prepare the surfaces for SEM imaging, micro-CT scanning, and nano-indentation studies.

*
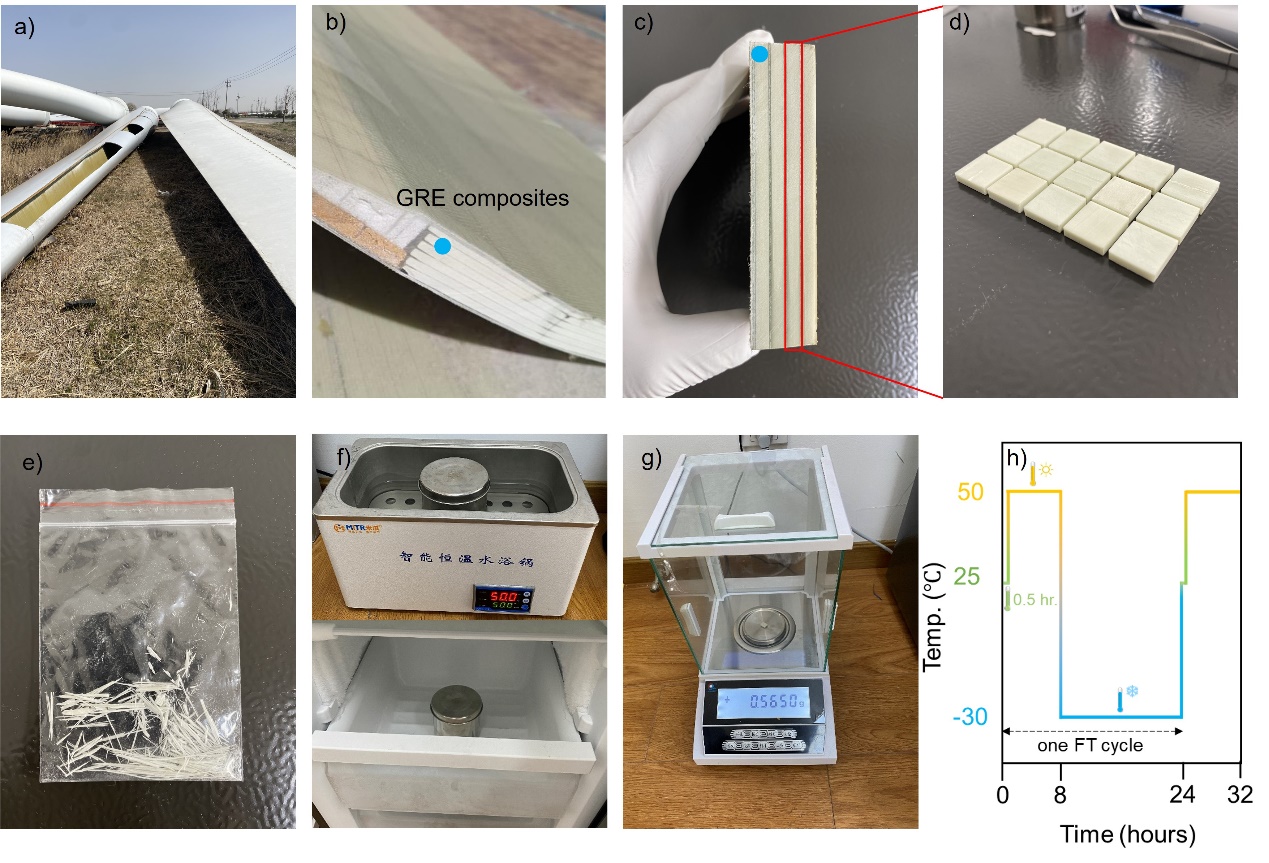
*

Supplementary Fig. S1. Overview of decommissioned wind turbine blade waste and sample preparation process. (a) Decommissioned wind turbine blade waste site. (b) Blade section showing balsa wood, foam, and glass fiber-reinforced epoxy (GRE) composites (blue circle). (c) Close-up of selected GRE composite layers (highlighted by the red rectangle). (d) Specimens cut from GRE layers for interface studies. (e) GRE composite shreds prepared for understanding crack propagation mechanism, elemental and chemical composition analysis before and after freeze-thaw treatment. (f) Lab-scale setup for freeze-thaw treatment. (g) Weighing balance used to measure specimen weight before and after freeze-thaw cycles. (h) Temperature profile and duration for one freeze-thaw cycle.

**Freeze-thaw Treatment Process**

Two freeze–thaw (FT) treatments were conducted in this study to address different experimental objectives, while maintaining consistent thermal cycling conditions across both treatments.

(1) The first FT treatment involved six GRE specimens and aimed to investigate interface weakening and crack initiation behavior. These specimens were immersed in natural water and subjected to thermal cycling in accordance with ASTM D6944-15. Each cycle consisted of 30 minutes at 25 °C, 8 hours at 50 °C, and 16 hours at –30 °C (Supplementary Figure S1f–h). The cycle was repeated continuously for three weeks. This duration was selected as the maximum threshold needed to induce interfacial crack development in GRE composites [65]. These specimens were used for scanning electron microscopy (SEM) and weight change analysis.

(2) A second set of GRE specimens, prepared and polished in parallel with the first batch *(Section 3.1.2)*, but not previously exposed to FT cycling, was subjected to a 10-day freeze–thaw treatment. This duration was chosen based on prior weight change data, which indicated peak water uptake and maximum structural degradation by Day 10 (section 3.1.2). These specimens were used for micro-computed tomography (micro-CT), nano-indentation, and post-treatment water quality analyses (pH, TOC, and microplastic release), using the same thermal cycling protocol as above to ensure consistency.

**GF-epoxy Separation Analysis**

To evaluate the properties of the collected wind turbine blade composites, various characterization techniques were employed to assess GF-epoxy separation efficiency including scanning electron microscopy (SEM), weight change analysis, and micro-computer tomography (micro-CT). These methods provided a comprehensive understanding of the GF-epoxy separation mechanism and process efficiency.

***Scanning Electron Microscopy (SEM)***

SEM was used to visualize surface morphology and fiber–resin interface separation in GRE specimens subjected to the 3-week FT treatment. Specimens were coated with a thin gold layer for conductivity and imaged using a Thermo Scientific Apero 2 system (Det ETD-SE). For crack propagation mechanism analysis, a few shredded GRE specimens were exposed to the same FT conditions to investigate the GF-epoxy separation mechanism.

***Weight Change Monitoring***

The first batch of all GRE specimens prior to FT treatment were initially weighed using a high-precision balance (Puchun Company) with a tolerance of ±0.0001 g, and this initial weight was recorded as $W_{o}$. These specimens were then subjected to three weeks’ freeze-thaw thermal cycling. Following each freeze-thaw cycle, two weight measurements were taken: pre-thaw weight, recorded after thawing, and post-thaw weight, recorded after the warm water immersion phase. The freeze–thaw cycles continued until all three stages of the proposed GF–epoxy separation mechanism i.e. water ingress, interfacial crack initiation, and crack propagation were observed.

Due to structural variability across specimens, the median weight change was used as a representative value to analyze overall trends.

Pre-thaw and post-thaw weight changes were calculated using Eq. 1(i) [30] .

$W_{Pre/Post-thaw}=\left( \frac{W_{final}-W_{initial}}{W_{initial}} \right)\times100$

***Micro-Computed Tomography (micro-CT)***

To assess glass fiber–epoxy (GF-epoxy) interfacial separation and crack propagation, micro-CT analysis was performed on representative GRE specimens before and after ten days of freeze–thaw treatment. Day 10 was selected based on earlier weight change data, which showed peak water uptake and maximum structural deformation by this stage. Scanning was conducted using a Tianjin Sanying Nano Voxel system with a spatial resolution ≤3 µm. Both pre- and post-treatment samples were imaged under identical conditions using a 180 kV, 200 µA nano-focus X-ray tube to ensure comparability. Over 1700 slices per specimen were acquired at a resolution of 1920 × 1080 pixels and reconstructed into 3D volumes. Post-scan analysis was carried out using AVIZO 3D (version 2022) to quantify average crack volume, porosity, and changes in pore/crack diameter distribution.

**Material Characterization**

***Energy Dispersive X-ray Spectroscopy (EDS)***

Energy Dispersive X-ray spectroscopy (EDS) was utilized for shredded GRE composites before and after three weeks of freeze-thaw to analyze the retention of glass fiber’s core elements by using Gemini 2 model, ZEISS.

***Fourier Transform Infrared Spectroscopy (FTIR)***

FTIR spectroscopy was used to identify the retention of functional groups and chemical bonds present in the glass fibers and epoxy before and after three weeks of freeze-thaw treatment. Spectra were recorded over a wavenumber range of 4000 to 500 cm⁻¹.

***Thermogravimetric Analysis (TGA)***

Thermogravimetric analysis (TGA) was conducted to evaluate the thermal stability and decomposition behavior of the GRE composites before and after three weeks of freeze-thaw treatment. The analysis was performed using a TGA-55 model (TA Instruments), with test samples from whole GRE composites (untreated and three weeks’ FT treated) heated from room temperature to 800°C at a rate of 10°C/min in a nitrogen atmosphere.

**Nano-mechanical Properties Testing**

Nanoindentation is widely regarded as a reliable non-destructive method for evaluating the mechanical behavior of small-diameter fibers where conventional testing is impractical [66,67]. In the present study, individual glass fiber tensile testing was not feasible due to the main focus on GF–epoxy interface weakening rather than individual glass fibers reclamation. Therefore, in situ nano-indentation mechanical testing was performed on 10-days FT treated GRE specimens. Nano-indentation was conducted using an Anton Paar NHT2 system (Austria) equipped with a Berkovich diamond tip at a maximum load of 1000 mN in fixed-force mode. The Oliver and Pharr method [67] was applied to calculate hardness (*H*), maximum indentation depth $h_{max}$, Hardness ($H$), and Reduced Young’s Modulus ($E_{r}$). Five fibers (diameter ≈ 17 ± 1 µm) were selected for each condition. Data processing was performed using Gwyddion software. To ensure meaningful comparison, five visually selected embedded glass fibers with diameters of ~17± 1 µm were indented before and after FT treatment.

**Post Freeze-thaw Water Quality Assessment**

To evaluate the environmental compliance of the FT recycling method, effluent water from the 10-day FT treatment was analyzed for microplastic content, pH, and total organic carbon (TOC). After FT treatment, GRE specimens were removed and settled epoxy debris was filtered using 5 μm pore qualitative filter paper (Delvstlab). Filtered particles were examined via SEM, and 300 particles were analyzed using Fiji software to determine size distribution. Water pH was measured following GB/T 5750.4-2023 at 25 °C, TOC was measured by using a Shimadzu TOC-L CPH analyzer. Baseline comparisons were made using influent water sourced from the institute’s filtered drinking water system.

**Supplementary Note S2: Pre-Existing Micro-Cracks in Wind Turbine Blades**

As shown in Supplementary Figure S1, micro-cracks at the fiber–resin interface in the GRE (glass fiber reinforced epoxy) composite are not detectable at low (20 µm) or medium (5 µm) magnification. Only at high magnification (1 µm) are such interfacial de-bonding features observed. Importantly, these defects are not uniformly distributed; only a limited number of fibers exhibit visible de-bonding. However, these localized micro-cracks may act as initiation points for interfacial crack propagation, potentially extending to adjacent fibers and contributing to a progressive fiber–resin separation mechanism under freeze–thaw cycling.


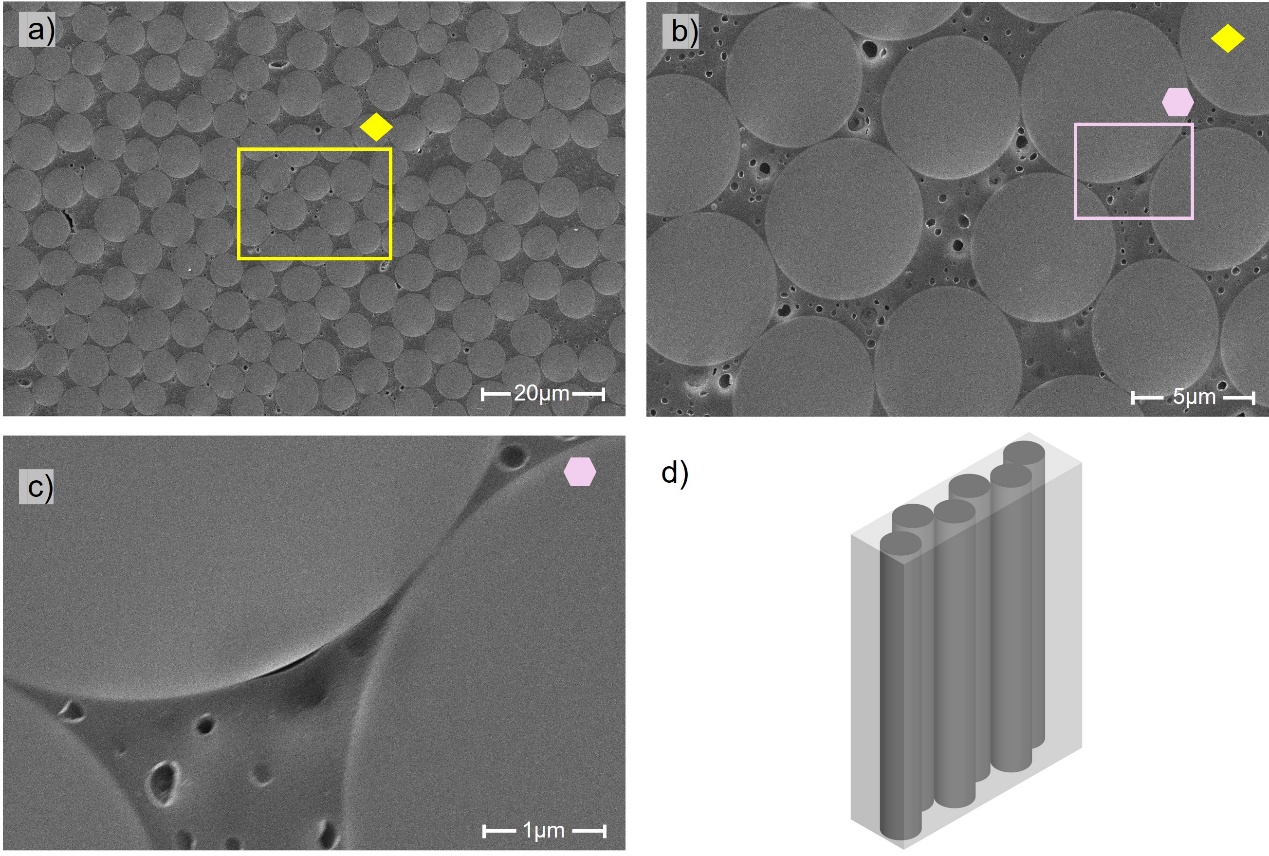


Supplementary Figure S2 Multiscale SEM and schematic of GRE composites.(a–c) SEM images at increasing magnifications reveal pre-existing micro-cracks at the glass fiber–epoxy (GF–epoxy) interface. While the overall structure appears intact, interfacial defects are visible only at high resolution. (d) Schematic showing the transverse fiber-aligned architecture of the GRE composite.

**Supplementary Note S3: Localized Epoxy Separation**

Localized morphological changes in GRE composites before and after treatment revealed visible cracks, epoxy fracture, and fiber-resin de-bonding (Supplementary Fig. S3). The initial state of the GRE composite shows glass fibers uniformly embedded in the epoxy resin matrix. The fiber-resin interface appears well-bonded, with no visible signs of separation or damage. This strong adhesive bond contributes to the structural integrity of the composite (Supplementary Fig. S3ab). In contrast, after three weeks of freeze-thaw cycling, significant changes are evident in the GRE composite, including interfacial debonding in areas prone to cracking, with evident separation of epoxy from the glass fiber surfaces (Supplementary Fig. S3cd). Matrix cracking was also observed throughout the resin, likely due to differential thermal expansion and contraction during the freeze-thaw process. These observations confirm that freeze-thaw treatment induces debonding and crack propagation at the fiber-resin interface, which is a critical barrier in recycling composite waste from decommissioned wind turbine blades.


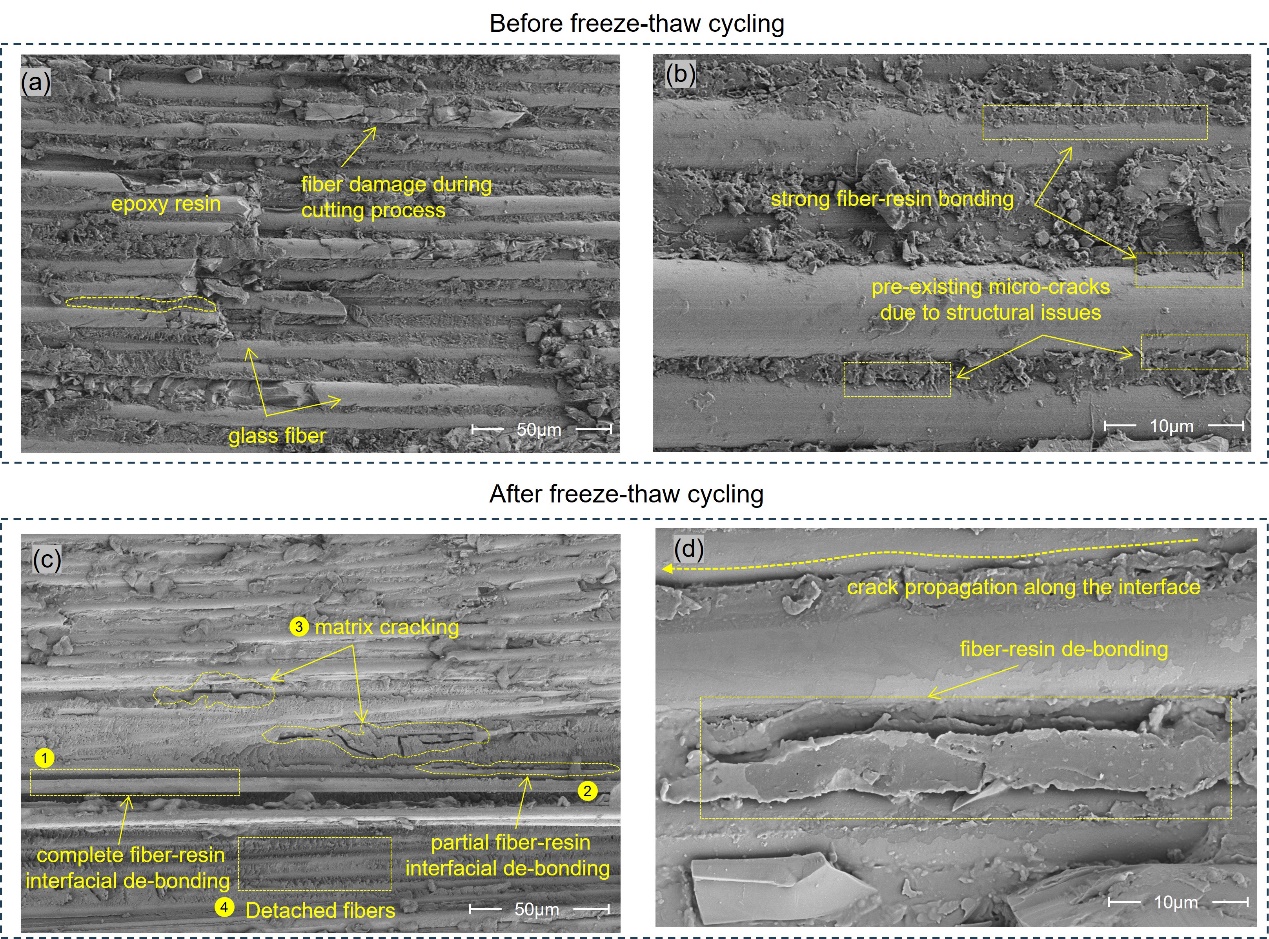


Supplementary Fig. S3. SEM images of GRE composites before and after freeze-thaw treatment
